# Automatable Distributed Regression Analysis of Vertically Partitioned Data Facilitated by PopMedNet: Feasibility and Enhancement Study

**DOI:** 10.2196/21459

**Published:** 2021-04-23

**Authors:** Qoua Her, Thomas Kent, Yuji Samizo, Aleksandra Slavkovic, Yury Vilk, Sengwee Toh

**Affiliations:** 1 Department of Population Medicine Harvard Medical School Boston, MA United States; 2 Harvard Pilgrim Health Care Institute Boston, MA United States; 3 Department of Statistics Pennsylvania State University University Park, PA United States

**Keywords:** distributed regression analysis, distributed data networks, privacy-protecting analytics, vertically partitioned data, informatics, data networks, data

## Abstract

**Background:**

In clinical research, important variables may be collected from multiple data sources. Physical pooling of patient-level data from multiple sources often raises several challenges, including proper protection of patient privacy and proprietary interests. We previously developed an SAS-based package to perform distributed regression—a suite of privacy-protecting methods that perform multivariable-adjusted regression analysis using only summary-level information—with horizontally partitioned data, a setting where distinct cohorts of patients are available from different data sources. We integrated the package with PopMedNet, an open-source file transfer software, to facilitate secure file transfer between the analysis center and the data-contributing sites. The feasibility of using PopMedNet to facilitate distributed regression analysis (DRA) with vertically partitioned data, a setting where the data attributes from a cohort of patients are available from different data sources, was unknown.

**Objective:**

The objective of the study was to describe the feasibility of using PopMedNet and enhancements to PopMedNet to facilitate automatable vertical DRA (vDRA) in real-world settings.

**Methods:**

We gathered the statistical and informatic requirements of using PopMedNet to facilitate automatable vDRA. We enhanced PopMedNet based on these requirements to improve its technical capability to support vDRA.

**Results:**

PopMedNet can enable automatable vDRA. We identified and implemented two enhancements to PopMedNet that improved its technical capability to perform automatable vDRA in real-world settings. The first was the ability to simultaneously upload and download multiple files, and the second was the ability to directly transfer summary-level information between the data-contributing sites without a third-party analysis center.

**Conclusions:**

PopMedNet can be used to facilitate automatable vDRA to protect patient privacy and support clinical research in real-world settings.

## Introduction

Researchers often have to pool data from multiple sources for their studies. One common scenario is to combine data from multiple distinct cohorts of patients to achieve sufficient statistical power, especially in studies where the exposure or outcome of interest is rare. Another scenario is when important variables such as exposures, outcomes, or confounders are available from multiple data sources. However, physical pooling of patient-level data dispersed across multiple data sources often raises several concerns, including ownership of the data, unapproved use of the transferred data, and proper protection of patient privacy and proprietary interests of the data-contributing sites [1-5].

In most studies that analyze patient-level data from multiple sites, researchers can remove direct patient identifiers (eg, name, social security number) before sharing the data. It is also possible to relativize certain data attributes such as dates (eg, by setting the cohort entry date as time zero and converting all dates to numerical values relative to the time zero), perturb data attributes that may be used to reidentify patients (eg, rare covariates or laboratory values), or encrypt the deidentified patient level. However, these data manipulation techniques may not be feasible in certain studies and do not always guarantee adequate levels of privacy protection, which may deter collaboration and data sharing.

**Figure 1:**
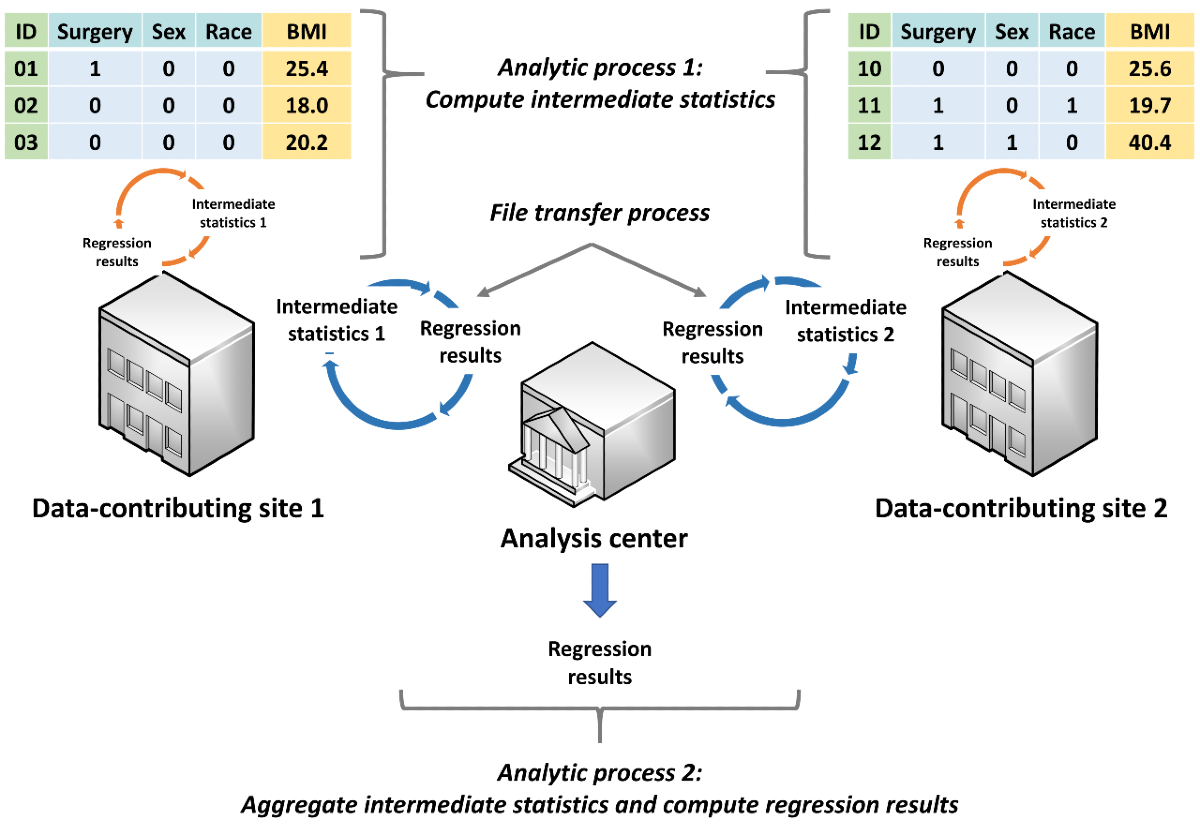
Distributed regression analysis in horizontally partitioned data environments. In this hypothetical example, surgery, sex, and race are the independent variables, while BMI is the dependent variable. Both data-contributing sites have the same set of variables.

**Figure 2:**
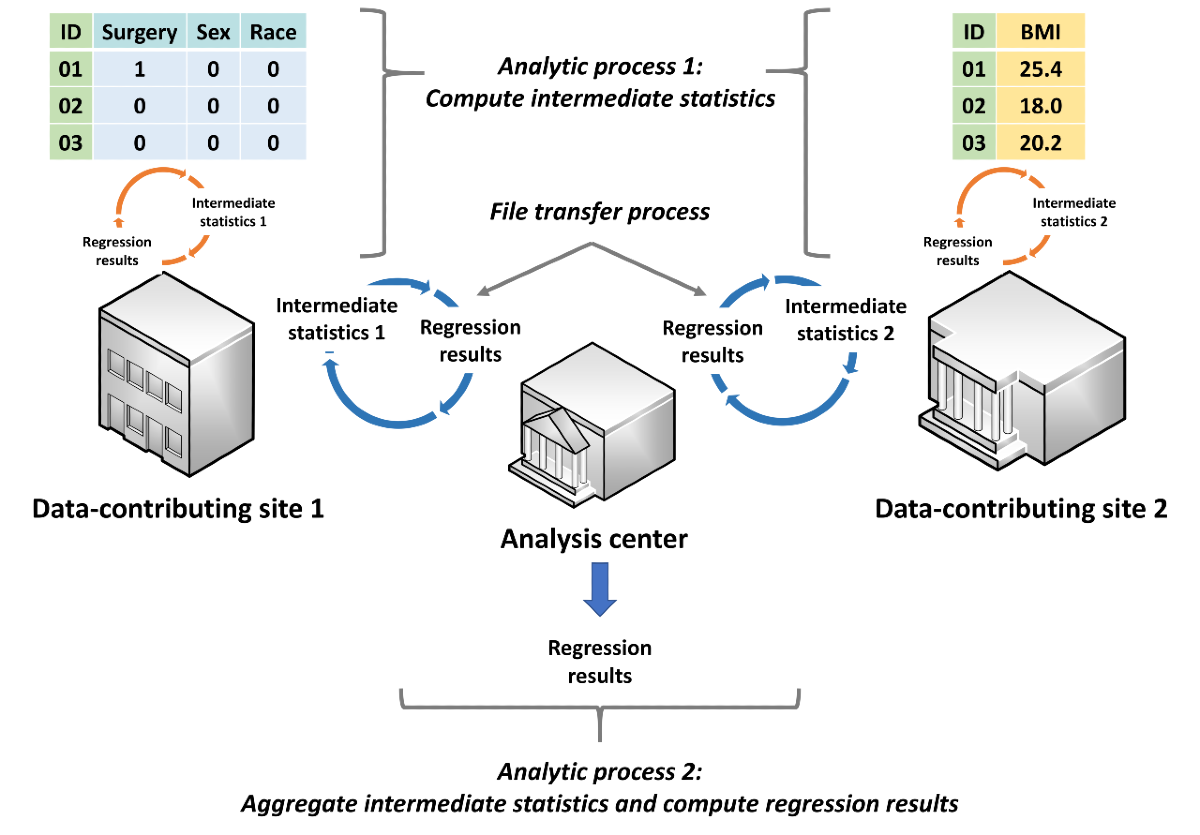
Distributed regression analysis in vertically partitioned data environments. In this hypothetical example, surgery, sex, and race are the independent variables, while BMI is the dependent variable. Data-contributing site 1 has data on the independent variables, while data-contributing site 2 has data on the dependent variable. Data-contributing site 2 can also have additional independent variables, as another variant of vertically partitioned data environments.

A number of privacy-protecting analytic methods have been developed to complement available data manipulation techniques. These methods, including meta-analysis of site-specific effect estimates and methods that leverage confounder summary scores, generally only require data-contributing sites to share summary-level information, thereby offering better privacy protection [6-8]. However, most methods were developed to analyze horizontally partitioned data (Figure 1), a setting where distinct cohorts of patients with the same data attributes are available in multiple data-contributing sites [9,10]. There are few valid and practical privacy-protecting methods to analyze vertically partitioned data (Figure 2), a setting where the data attributes of one distinct cohort of patients are available in two or more data-contributing sites.

**Figure 3:**
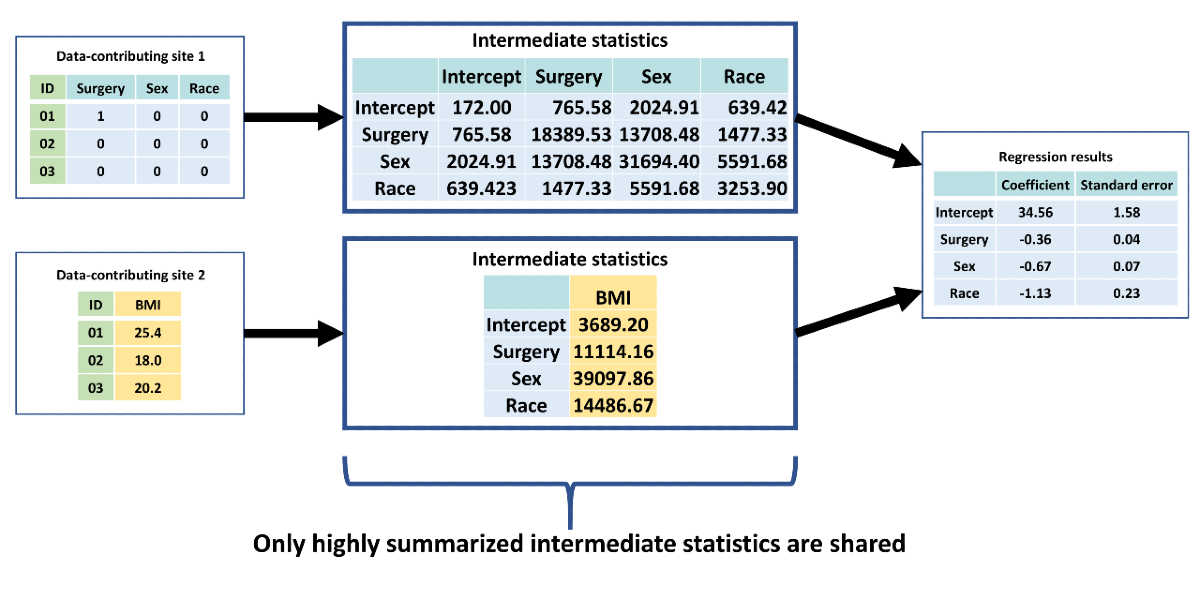
A hypothetical example of intermediate statistics shared by data-contributing sites in distributed linear regression analysis of vertically partitioned data. In this hypothetical example, surgery, sex, and race are the independent variables, while BMI is the dependent variable. Data-contributing site 1 has data on the independent variables, while data-contributing site 2 has data on the dependent variable.

A promising privacy-protecting analytic method for both horizontally and vertically partitioned data is distributed regression, a suite of methods that perform multivariable-adjusted regression analysis with only highly summarized intermediate statistics of the patient-level data from the data-contributing sites (Figure 3) [11-13]. We have previously developed an SAS-based distributed regression analysis (DRA) package integrated with PopMedNet, an open-source file transfer software [14-17], to perform automatable distributed regression within horizontally partitioned data environments (horizontal DRA [hDRA]). We successfully tested the application in a real-world setting [18]. The feasibility of using PopMedNet to facilitate DRA with vertically partitioned data (vertical DRA [vDRA]) has not been evaluated. In this article, we describe the feasibility of using PopMedNet and enhancements to PopMedNet to facilitate automatable vDRA to protect patient privacy and support clinical research in real-world settings.

## Methods

### Required Processes for Distributed Regression Analysis

Distributed regression in both horizontally and vertically partitioned environments requires two distributed analytic processes and a file transfer process (Figures 1 and 2) [11-13]. The first analytic process, which occurs at the data-contributing sites, involves computing and sharing the intermediate statistics of patient-level data with other data-contributing sites or a semitrusted third-party analysis center. The second analytic process, which occurs at the analysis center, involves aggregating the intermediate statistics and computing regression parameter estimates, standard errors, model fit statistics, and any necessary graphs (collectively called regression results hereafter). For some regression model types (eg, logistic and Cox proportional hazards), these two processes are iterative and continue until a prespecified convergence criterion is met or a prespecified maximum number of iterations is reached. When one of these prespecified conditions is fulfilled, the regression results are retained as the final results and the analysis is completed. Otherwise, the updated regression results are shared with the data-contributing sites and used to further refine the intermediate statistics. Manual transfer of the intermediate statistics can be cumbersome and error-prone. Alternatively, a semiautomated or fully automated file transfer process can be used to facilitate the iterative transfer of the intermediate statistics and regression results between the participating parties.

### Implementation of hDRA Using SAS and PopMedNet

We have previously developed an SAS-based DRA package to perform the two distributed analytic processes for hDRA in a real-world setting [11,12]. This package requires an analysis center to facilitate its execution. We developed an automatable file transfer process during the execution of the DRA package by enhancing PopMedNet [1], an open-source file transfer software currently used by several large, distributed data networks, to securely transfer files through a locally installed Microsoft Windows application known as a DataMart Client using HTTPS/SSL/TLS connections [19]. PopMedNet ensures that only approved data queries are requested. Authenticated data-contributing sites are only able to transfer files to the analysis center and not to each other. All file transfers are managed by a web-based portal accessible only by the analysis center.

We integrated the SAS-based DRA package with PopMedNet to create a DRA application. The DRA application can perform distributed linear, logistic, and Cox proportional hazards regression analysis [18]. We were able to compute regression results through DRA that were precise (difference <10-12) to the regression results from the corresponding pooled patient-level data analyses using standard SAS procedures. We were also able to generate model fit graphics that were similar to the graphics obtained from the corresponding patient-level regression analysis using standard SAS procedures. With a sample size of 5452 patients, each regression model type required less than 20 minutes to complete, with the file transfer time accounting for approximately 90% of the total execution time. The hDRA application did not require participating data-contributing sites to install new software or substantially modify their hardware configuration because PopMedNet and SAS were already installed on their systems.

### Feasibility of Performing vDRA Using PopMedNet

**Figure 4:**
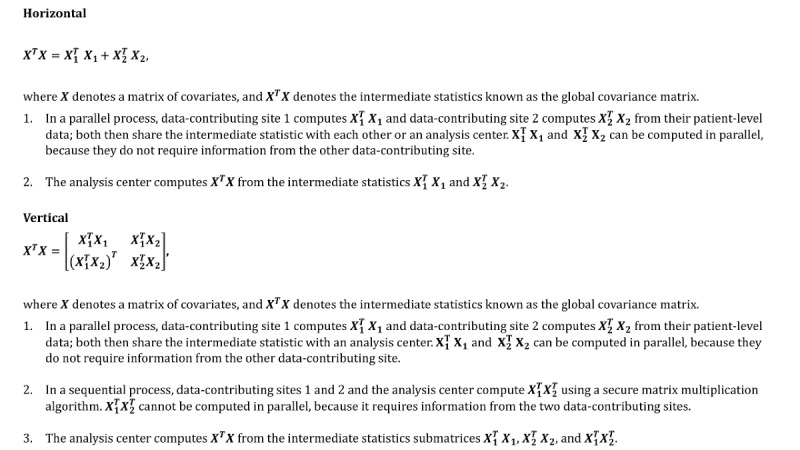
Processes required to perform distributed linear regression in horizontally or vertically partitioned data environments with two data-contributing sites and an analysis center.

The distributed matrix computations are more complex and computationally more intensive in vDRA than in hDRA [20]. Data-contributing sites are also required to share more granular summary-level information in vDRA, leading to larger files being transferred. In hDRA, the modeling process is comprised of data-contributing sites computing the intermediate statistics and the analysis center aggregating the intermediate statistics and computing the regression results. Most of these computations can be completed in parallel. In contrast, numerous parts of the vDRA modeling process are sequential. Specifically, the data-contributing sites must first compute components of the intermediate statistics and then compute the intermediate statistics from the components. For example, to compute the intermediate statistic known as the global covariance matrix (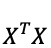, where 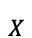 denotes a matrix of covariates) for vDRA, the data-contributing sites must first compute their site-specific covariance matrix (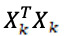, where *k* denotes data-contributing site *k*) and then the off-diagonal components (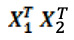) of the global covariance matrix using a sequential and secure matrix multiplication algorithm (Figure 4) [20]. Once all of the components are computed, the analysis center then aggregates the intermediate statistics and computes the regression results.

PopMedNet was not designed to optimally support these differences. Thus, we gathered the statistical and informatic requirements of vDRA and performed a feasibility analysis of using PopMedNet to facilitate vDRA with an automatable file transfer process. We used the results of this analysis to identify, develop, and implement enhancements to PopMedNet to improve its technical capability to facilitate automatable vDRA. As guiding principles, we required enhancements to support execution times that lasted for only a few hours, to not disrupt existing PopMedNet workflows, to be developed and implemented with minimal to moderate effort at the data-contributing sites, and to not lead to major modifications to hardware configurations or new software installations at the data-contributing sites.

## Results

### Findings From Assessment of the Existing PopMedNet Functionalities to Facilitate Automatable vDRA

With the prior enhancements to PopMedNet (version 6.7) to facilitate automatable hDRA, we had the necessary technical infrastructure to perform automatable vDRA. However, vDRA would be limited to analysis of small cohort sizes and regression models with few covariates. As described above, vDRA requires matrix computations that are more complex and computationally more intensive than hDRA, data-contributing sites to share more granular information and files of larger sizes, and a modeling process that is mostly sequential. These differences would increase computation time and file transfer time, which would lead to considerably longer execution times (Table 1). Moreover, executing vDRA with a large cohort of patients or a large number of covariates would further increase execution time and render the PopMedNet configuration developed for hDRA impractical. Additional enhancements to PopMedNet were necessary to ensure that DRA is a feasible analytic option for vertically partitioned data.

**Table 1:** Differences between horizontal and vertical distributed regression analysis and their impacts on execution time.

	Distributed regression analysis			
Component of analysis	Horizontal	Vertical	Impact on execution time in vertical distributed regression analysis^a^	
Computation sequence^b^	Computes intermediate statistics Computes regression results^c^	Computes components of the intermediate statistics Computes intermediate statistics Computes regression results^c^	Increases computation and file transfer times	
Computation process	Most computations can be completed in a parallel process	Most computations require a sequential process	Increases computation and file transfer times	
File transfer sizes	Kilobytes to megabytes [18]	Kilobytes to infinity^d^	Increases file transfer times	
Dimension of an example matrix transfer^e^	*p* × *p*	*n* × *n*	Increases file transfer times	

^a^Execution time in horizontal distributed regression analysis serves as the baseline.

^b^Sequence of computations required to compute regression results; the sequence is iterative for some regression model types.

^c^Include regression parameter estimates, standard errors, model fit statistics, and model fit graphics.

^d^File sizes increase as cohort size or number of covariates in the regression model increases.

^e^*p* is the number of regression model covariates; *n* is the number of observations at each data-contributing site.

### Implemented Enhancements to PopMedNet to Enable Automatable and Efficient Implementation of vDRA

We identified and implemented two enhancements to PopMedNet to improve its technical capability to facilitate automatable vDRA in the real-world setting. These enhancements decrease execution time, require only minimal or moderate development and implementation efforts, do not disrupt existing PopMedNet workflows, and do not require data-contributing sites to modify their hardware configurations.

**Figure 5:**
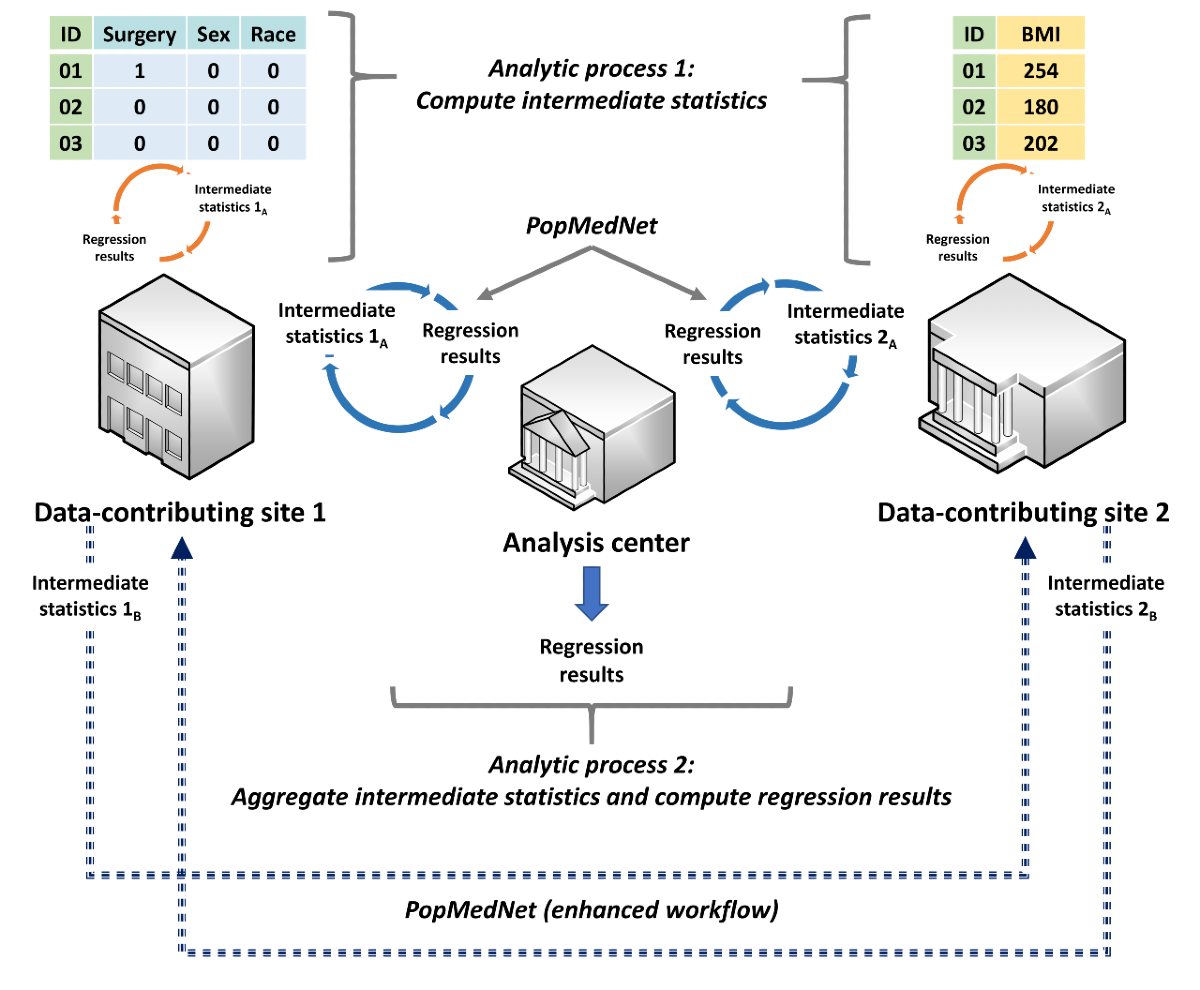
Enhanced PopMedNet workflow to enable automatable distributed regression analysis in vertically partitioned data environments. In this hypothetical example, surgery, sex, and race are the independent variables, while BMI is the dependent variable. Data-contributing site 1 has data on the independent variables, while data-contributing site 2 has data on the dependent variable.

First, we enhanced PopMedNet to allow simultaneous upload and download of multiple files. The previous version of PopMedNet only allowed one file to be uploaded and downloaded at a given time. Concurrent transfer of multiple files decreases file transfer time. Second, we enhanced PopMedNet to allow direct file transfers between data-contributing sites (Figure 5). Previously, PopMedNet did not allow direct file transfers between data-contributing sites. File transfer was only possible between the analysis center and the data-contributing sites; any files to be shared between data-contributing sites first had to be transferred through the analysis center. This design aimed to reduce the potential risk of two or more data-contributing sites colluding against the other sites to derive information about specific patients using the summary statistics, but it doubled the file transfer times. Allowing direct file transfers between the data-contributing sites would enable vDRA algorithms that are simpler, share less granular information, share smaller files, and require fewer file transfers. It would also allow more computations to be done in parallel, which would decrease computation and file transfer times.

To minimize the risk of collusion under the enhanced file transfer process, we implemented a trust matrix, where the analysis center can prespecify and govern the file transfer process between data-contributing sites. Only the analysis center will have access and permission to modify the trust matrix. Any data-contributing sites that violate the trust model will prompt PopMedNet to terminate the analysis.

## Discussion

### Principal Findings

PopMedNet can facilitate the execution of automatable vDRA. We identified and implemented two enhancements to PopMedNet that improved its technical capability to facilitate vDRA in real-world settings. The first enhancement was concurrent uploading and downloading of multiple files and the second involved enhancing the PopMedNet trust model to allow certain prespecified and preapproved file transfer processes between the data-contributing sites. Both enhancements decrease the file transfer and computation times needed to perform vDRA, which limit connectivity issues (eg, firewall timeouts, network connections, and connections to the internet) as a barrier to performing vDRA in real-world settings. Connectivity may seem inconsequential with high-speed internet and high-performing computers, but issues of connectivity are compounded when there are multiple parties participating in a regression analysis that requires numerous iterations and file transfers.

### Other Research in vDRA

There have been efforts to make vDRA a practical analytic option in real-world settings. Li and colleagues [3] developed VERTIcal Grid lOgistic regression (VERTIGO) to perform distributed logistic regression analysis in vertically partitioned data. Dai and colleagues [4] recently developed VERTICOX to perform distributed Cox proportional hazards regression analysis in vertically partitioned data. Both studies found that cohort size influenced the operational performance of their methods. Similar to the findings in our feasibility analysis, Li and colleagues [3] concluded that vDRA performed with VERTIGO was limited to analyzing relatively small cohort sizes. These authors identified several potential solutions to improve the operational performance of VERTIGO, including performing the matrix computations on graphic processing units, parallelizing the matrix computations on multiple cores or machines, and dividing the matrix computations into smaller parts (a divide-and-conquer strategy). Performing vDRA on graphic processing units may require new hardware, which would violate our guiding principles. We explored parallelizing the matrix computations in our feasibility analysis but concluded that it would require meaningful changes to the hardware configuration and installation of new software at the data-contributing sites. We explored implementing a similar divide-and-conquer strategy in the design of our vDRA algorithms by first horizontally partitioning the vertically partitioned data sets into smaller blocks and then performing the required matrix computations within the blocks. This design should decrease computation times because the computations are completed with smaller matrices.

### Limitations

Our two enhancements improved PopMedNet’s technical capability to facilitate vDRA. However, there are several factors that may limit the use of PopMedNet to enable automatable vDRA in real-world settings. First, real-world implementation of PopMedNet-supported vDRA will be driven by the degree of automation between the participating data-contributing sites and the file transfer speed of the slowest site. Numerous parts of the vDRA modeling process are executed sequentially. Thus, if a site performs vDRA with a manual file transfer process or has a slow file transfer speed, the overall execution time will be limited by the response time of the slowest site. This may deter data-contributing sites from using vDRA or PopMedNet to analyze data from multiple data sources.

Second, we could only implement enhancements to PopMedNet that adhered to our guiding principles, which were developed based on existing PopMedNet users. There may be unforeseen challenges that require major enhancements or changes to the PopMedNet topology and infrastructure if vDRA were to be conducted in other data-contributing sites with different software and hardware configurations.

Third, the acceptance of automatable vDRA and enhanced PopMedNet capabilities by data-contributing sites should not be overlooked. In our previous experience, data-contributing sites were reluctant to use the fully automated PopMedNet workflow to facilitate hDRA. It was only after an initial roll-out phase with the manual and semiautomated workflows and confirmation that the intermediate statistics did not contain identifiable patient information that they were willing to experiment with the fully automated PopMedNet workflow. With vDRA requiring the transfer of more granular information and longer computation and file transfer times than hDRA, some data-contributing sites may require a similar roll-out phase to build trust and acceptance of automatable vDRA.

Fourth, we chose to build upon our previous work and enhanced PopMedNet to facilitate vDRA. This may limit our vDRA package to organizations who use PopMedNet as their file transfer software. However, PopMedNet is currently used by several large distributed data networks, including the Sentinel System [14], the National Patient-Centered Clinical Research Network [15], and the National Institutes of Health Health Care Systems Research Collaboratory [16]. These networks have established infrastructure (eg, harmonized data, data use agreements, governance) and processes that can streamline the use of vDRA to analyze data from multiple data sources. Thus, these networks can readily implement PopMedNet-supported hDRA and vDRA.

### Future Work

With the enhancements to PopMedNet, we have started implementing the divide-and-conquer vDRA algorithms that leverage PopMedNet’s new functionality to transfer files between data-contributing sites. We are also exploring enhancements to the vDRA algorithms to reduce the number of files needed to be transferred. Reducing the number of files transferred will decrease the overall file transfer time and make vDRA a practical analytic option in real-world settings. To offer better privacy protection, we are only implementing vDRA algorithms that limit the potential for back calculations. We will explore the feasibility of combining our vDRA algorithms with data manipulation techniques, such as perturbation and encryption, to provide additional layers of protection [5]. We plan to integrate these vDRA algorithms with PopMedNet to create a vDRA application and test it with real-world data.

### Conclusion

PopMedNet can be used to facilitate automatable vDRA. We have implemented two enhancements to the PopMedNet workflow to improve its technical capability to facilitate vDRA in real-world settings. PopMedNet has the potential to increase clinical research and collaboration across multiple data-contributing sites while protecting patient privacy and proprietary interests of the data-contributing sites.

## Abbreviations

DRA: distributed regression analysis
hDRA: horizontal distributed regression analysis
vDRA: vertical distributed regression analysis
VERTIGO: VERTIcal Grid lOgistic regression
